# Total Solution-Processed Zr: HfO_2_ Flexible Memristor with Tactile Sensitivity: From Material Synthesis to Application in Wearable Electronics

**DOI:** 10.3390/s25206429

**Published:** 2025-10-17

**Authors:** Luqi Yao, Yunfang Jia

**Affiliations:** College of Electronic Information and Optical Engineering, Nankai University, Tianjin 300071, China; 2120240469@mail.nankai.edu.cn

**Keywords:** ferroelectric memristor, HZO film, total solution-processed method, interfacial oxygen competition, wearable electronics

## Abstract

In the pursuit of advanced non-volatile memory technologies, ferroelectric memristors have attracted great attention. However, traditional perovskite ferroelectric materials are hampered by environmental pollution, limited applicability, and the complexity and high cost of conventional vacuum deposition methods. This has spurred the exploration of alternative materials and fabrication strategies. Herein, a flexible Pt/Zr: HfO_2_ (HZO)/graphene oxide (GO)/mica memristor is successfully fabricated using the total solution-processed method. The interfacial oxygen competition mechanism between the HZO layer and the GO bottom electrode facilitates the formation of the HZO ferroelectric phase. The as-prepared device exhibits a switching ratio of approximately 150 and can maintain eight distinct resistance levels, and it can also effectively simulate neural responses. By integrating the ferroelectric polarization principle and the piezoelectric effect of HZO, along with the influence of GO, the performance variations of the as-prepared device under mechanical and thermal influences are further explored. Notably, Morse code recognition is achieved by utilizing the device’s pressure properties and setting specific press rules. The as-prepared device can accurately convert and store information, opening new avenues for non-volatile memory applications in silent communication and promoting the development of wearable electronics.

## 1. Introduction

Ferroelectric non-volatile memristors have garnered significant attention due to their low power consumption [1,2], multi-state memories [3], and stable switches among multiple memory states [4] compared to other non-volatile memristors. It is believed that these advantages originate from the asymmetric crystal structure of ferroelectric materials, which can induce self-polarization and produce various resistance states under the control of external electric fields [5]. Currently, there are lots of contributions about ferroelectric memristors, for example, in terms of storage performance, achieving operating speeds as low as 300 ps and durability over 10^9^ cycles [1], up to 32 different resistance states [3], etc. Regarding applications, significant advances have been made in image recognition using neural networks with ferroelectric memristors as synaptic nodes [6,7,8]. Additionally, ferroelectric memristors have also been applied in tactile and visual bionics [9,10,11]. Due to their excellent thermal and chemical stability, a large number of flexible ferroelectric neuromorphic devices have been reported recently to meet the growing demand for more functional wearable electronic devices [12,13,14,15]. Although ferroelectric memristors show excellent potential for development, they are still in the evolving stage, and low-cost and flexible ferroelectric materials, together with the methods of device fabrication, are the issues that should be addressed regarding their progression. Traditional perovskite ferroelectric materials, especially Pb (Zr, Ti), O_3_, and BiFeO_3_, suffer from the volatilization of Pb and Bi at high temperatures, which may lead to potential environmental contamination [16,17]. Meanwhile, the relatively small bandgap of perovskite ferroelectric materials limits their applicability in harsh conditions, such as high-temperature and high electric fields [16]. The discovery of ferroelectricity in Si-doped HfO_2_ ultrathin films in 2011 provided a promising solution to tackle the aforementioned issues associated with conventional perovskite ferroelectric devices [18]. In recent years, ferroelectric hafnium-based thin films (Zr:HfO_2_ (HZO)) have stood out in the fields of bionic synapses and flexible wearable devices due to their lead-free and non-toxic nature, large bandgap (5–6 eV), and excellent ferroelectric properties [5,19,20]. At present, the predominant methods for fabricating HZO thin films rely on vacuum deposition techniques, such as pulsed laser deposition [21,22], atomic layer deposition [23,24], and chemical vapor deposition [25]. Especially when responding to the demands of flexible applications, film transfer strategies are mostly employed [26,27]. Nevertheless, these methods involve complex processes and high costs. Therefore, it is essential to propose a total solution-processed method for the fabrication of HZO memristors.

The total solution-processed technique is dominant in flexible electronics due to its cost-effectiveness and ease of operation [28,29,30,31]. On this basis, the solution-processed technique has also achieved lots of advancements in ferroelectric devices [32,33,34,35]. In particular, recent studies on solution-processed HZO film have provided technical guidance for developing low-cost flexible HZO devices, primarily addressing the two technological difficulties regarding film formation and the regulation of ferroelectric crystal structure [36,37,38]. As reported, a precursor film can be formed by spin-coating the mixed solution of hafnium isopropoxide and zirconium nitrate oxide hydrate in 2-methoxyethanol; after which, the two chemicals react during annealing to produce HZO with a ferroelectric phase [36,37]. Similarly, hafnium chloride and zirconium oxychloride octahydrate can also be used as precursors for coating on glass substrates with the aid of mixtures of ethylene glycol and acetonitrile; the crystalline structure of the resulting films could be tailored by adding an AlO_x_ capping layer to induce the ferroelectricity of HZO [38]. Though these contributions can only realize solution-processed HZO film, they also highlight the feasibility of the total solution-processed technique in flexible HZO devices. Specifically, there have been no reports to date on flexible HZO ferroelectric memristors, which poses a challenge for the growth of wearable ferroelectric devices.

**Figure 1:**
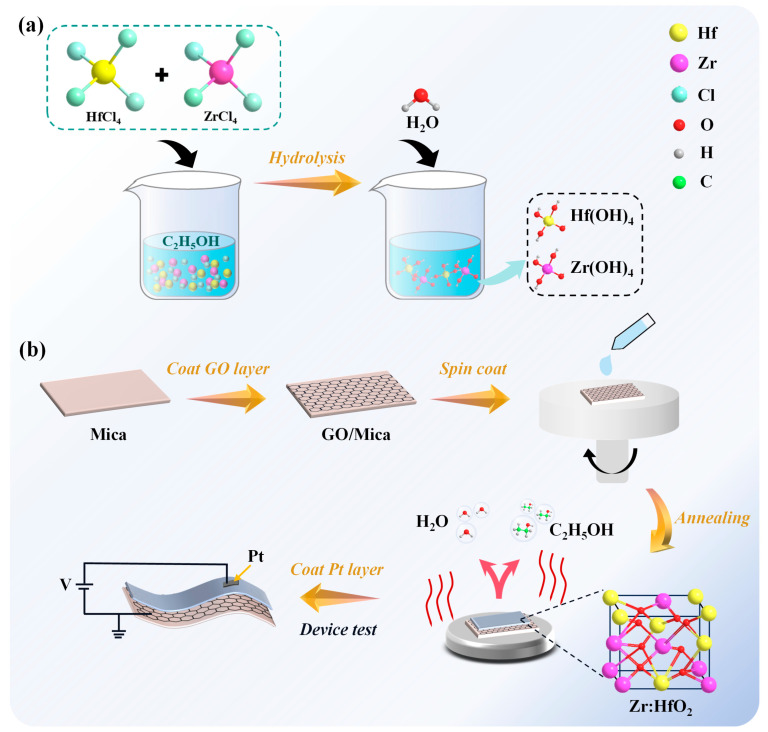
Schematic diagram of precursor solution preparation and device fabrication. (**a**) Scheme for the preparation process of HZO precursor solution. The precursors are HfCl_4_ and ZrCl_4_, and the solvent is an ethanol solution. (**b**) Scheme for the fabrication process of HZO-based flexible ferroelectric memristor and the device test.

Herein, a method of fabricating a total solution-processed flexible HZO ferroelectric memristor is proposed, as illustrated in Figure 1. In Figure 1a, the preparation process of the HZO precursor solution is depicted, where ethanol is selected as the solvent, offering enhanced safety compared with the toxic solvents mentioned above. Also, the polar nature of the ethanol solution facilitates the uniform dissolution of hafnium tetrachloride (HfCl_4_) and zirconium tetrachloride (ZrCl_4_), resulting in the generation of hafnium and zirconium ions, thereby providing a solid foundation for the subsequent hydrolysis reaction. The prepared HZO precursor solution is spin-coated onto the bottom electrode layer/substrate to form the precursor film, and the dehydration reaction occurs during the high-temperature annealing process, which leads to the formation of the mixture of hafnium oxide (HfO_2_) and zirconium oxide (ZrO_2_) film, as shown in Figure 1b. In this case, mica is chosen as the flexible substrate due to its much higher thermal resistance than normal flexible substrates (e.g., polyimide and polyester films), and it helps to avoid impurity contamination issues that may arise during the transfer process. Importantly, the interfacial oxygen competition between the HZO layer and the bottom electrode graphene oxide (GO) layer has facilitated the modulation of the crystal structure of HZO, promoting the formation of the ferroelectric phase. Compared to graphene oxide (GO) cured at 280 °C, the untreated graphene has fewer oxygen-containing groups, so the graphene conductive coatings form GO under a 280 °C high-temperature treatment (Figure S1). Based on the optimization of growth conditions, the fabricated HZO-based flexible ferroelectric memristor exhibits a switching ratio of ~150 and can stably maintain eight distinct levels. This indicates that the proposed devices could be an ideal candidate for a flexible multi-state HZO memristor; to the best of our knowledge, this is the first report about a total solution-processed HZO memristor. In terms of synaptic plasticity, two typical neuro-responses, which are paired-pulse facilitation (PPF) [39,40] and post-tetanic potentiation (PTP) [39,41,42], are simulated by the as-prepared memristors; the success of this experiment suggests that the proposed HZO-based memristor can be developed as an elemental pixel in artificial neural networks. Moreover, the performance of this memristor under mechanical and thermal influences has been comprehensively explored. Integrating the ferroelectric polarization principle and the piezoelectric effect of HZO, along with the influence of GO, reveals its adaptability to different environmental conditions. Interestingly, Morse code recognition is achieved by utilizing the device’s pressure properties. We hope this work can open new avenues for non-volatile memory applications in silent communication scenarios.

The remainder of this article is structured as follows. Section 2 introduces the experimental materials, device fabrication procedures, and testing methods. Section 3 tests the fabricated devices and discusses various characteristics of the devices. Section 4 outlines the key conclusions of this study.

The contributions of this study can be summarized as follows:

- A flexible Pt/Zr: HfO_2_ (HZO)/graphene oxide (GO)/mica memristor is proposed via the total solution-processed method.
- The interfacial oxygen competition mechanism between the HZO layer and the GO bottom electrode facilitates the formation of the HZO ferroelectric phase.
- The as-prepared device shows a switching ratio of ~150 and eight stable resistance levels, can effectively simulate neural responses, and exhibits good adaptability to mechanical deformations and temperature variations.

## 2. Methods

### 2.1. Materials

Hafnium tetrachloride powder (HfCl_4_, 99.99%) purchased from Shanghai Macklin Biochemical Technology Co., Ltd. (Shanghai, China). Zirconium tetrachloride powder (ZrCl_4_, 98%) purchased from Shanghai Meryer Biochemical Technology Co., Ltd. (Shanghai, China). Ethanol (C_2_H_5_OH, 99.8%) purchased from Shanghai Aladdin Biochemical Technology Co., Ltd. (Shanghai, China). Graphene (NCT-DR03) high-temperature coating purchased from Suqian Nakait New Material Technology Co., Ltd. (Suqian, China). Platinum paste (55L–2209) purchased from Shenzhen Saiya Electronic Paste Co., Ltd. (Shenzhen, China). Mica flakes purchased from Shenzhen Shunsheng Electronics Co., Ltd. (Shenzhen, China). Silicon wafer (N-type, crystal direction 100) purchased from Tianjin Ailian Electronic Technology Co., Ltd. (Tianjin, China).

### 2.2. Preparation of HZO Precursor Solution 

First, equal amounts of HfCl_4_ (0.15 g) and ZrCl_4_ (0.15 g) powder were mixed and dissolved in 4 mL ethanol solution with 2 mL deionized water and stirred vigorously using a magnetic stirrer (1500 rpm, 2 h). Then, the precursor solution was sonicated for 1 h by an ultrasonic cleaner to break up larger particles.

### 2.3. Preparation of Pt/HZO/GO/Mica Devices

First, the mica substrate was ultrasonically cleaned in acetone, ethanol, and deionized water sequentially for 10 min each. Then, the purchased graphene conductive coating was drop-coated onto the clean mica substrate and cured at 280 °C for 30 min to form GO. Detailed information on the analysis of oxygen-containing groups of untreated graphene is shown in Figure S1. Next, the HZO precursor solution was spin-coated onto the GO at a low speed of 500 rpm for 10 s and a high speed of 3000 rpm for 20 s. Subsequently, drying was carried out at 90 °C for 10 min, and the spin-coating and drying were repeated. Finally, a high-temperature annealing was performed at 600 °C for 2 h. Lastly, the Pt-conductive paste was uniformly dispensed onto the HZO film layer using a dispensing needle with an inner diameter of 0.25 mm.

### 2.4. Characterization and Measurement

The crystalline phase of the prepared HZO/GO/mica was characterized by X-ray diffraction (XRD, X’er Pro, Almelo, The Netherlands). The chemical elements and groups of HZO/GO were analyzed by X-ray photoelectron spectroscopy (XPS, Thermo Scientific ESCALAB 250Xi, Waltham, MA, USA). The microstructure and morphology of the prepared HZO/GO/mica were characterized by scanning electron microscopy and X-ray spectroscopy (SEM and EDS, JSM-7800F, Tokyo, Japan). All electrical measurements were performed using an Agilent semiconductor parameter analyzer (B2900A, Agilent Technologies, Inc., Santa Clara, CA, USA).

## 3. Results and Discussion

### 3.1. Characterization of the Solution-Processed HZO Films

**Figure 2:**
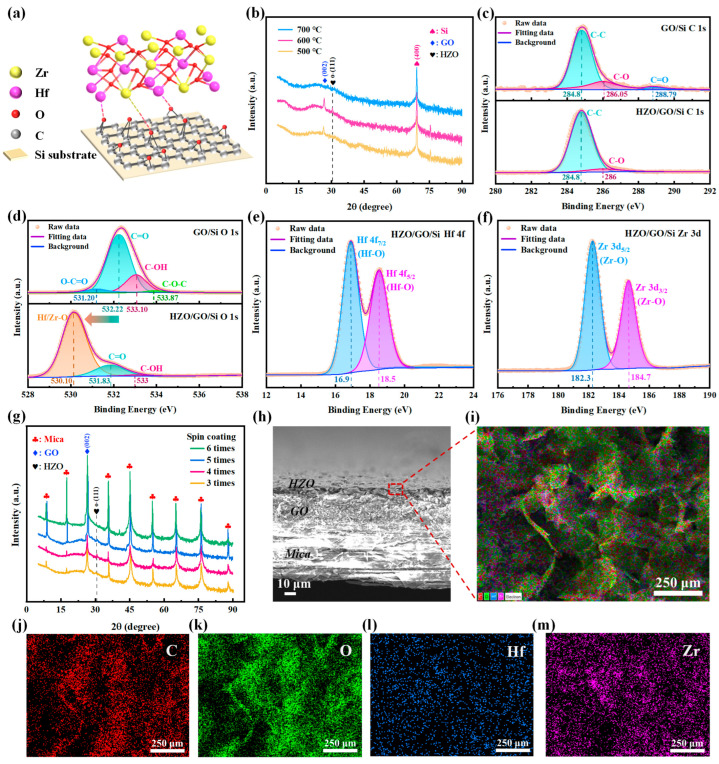
Characterization of crystalline phases, chemical elements, groups, and micromorphology of solution-processed HZO films. (**a**) Schematic representation of the HZO/GO/Si structure and the dynamic competition between the HZO and GO layers for oxygen atoms. (**b**) The XRD results of the HZO/GO/Si structure at 500 °C, 600 °C, and 700 °C. The peak at 26.4° is GO (002), the peak at 30.4° is HZO o- (111), and the peak at 69.3° is Si (400). (**c**) XPS core spectra for C 1s of GO/Si (the upper part) and HZO/GO/Si (the bottom part). (**d**) XPS core spectra for O 1s of GO/Si (the upper part) and HZO/GO/Si (the bottom part). (**e**) XPS core spectra for the Hf 4f of the Hf-O bond in HZO/GO/Si. (**f**) XPS core spectra for the Zr 3d of the Zr-O bond in HZO/GO/Si. (**g**) XRD results for the HZO/GO/mica structure at different spin-coating times (3, 4, 5, and 6 times). (**h**) SEM image of the cross-section of the HZO/GO/mica structure. The scale is 10 μm. (**i**) EDS mapping of the uppermost surface region marked by the red dashed line box in (**h**). The scale is 250 μm. (**j**–**m**) Spatial distribution and qualitative analyses for the C, O, Hf, and Zr elements of the layered HZO/GO. The scale is 250 μm.

To fabricate the total solution-processed HZO film, a GO-assisted HZO film on Si substrate was constructed, as illustrated in Figure 2a; its crystalline phases, chemical elements, and groups are thoroughly examined and presented in Figure 2b–f to optimize the annealing temperature and verify the GO-aiding function. First, the results of the X-ray diffraction (XRD) tests in Figure 2b reveal that the as-prepared HZO films’ crystalline phases depend on the annealing temperatures, which are 500 °C, 600 °C, and 700 °C. The peaks at 69.3° on three curves are believed to be generated by the diffraction of the Si (400) phase [43]. The formation of the metastable ferroelectric orthorhombic phase (i.e., the o-(111) phase) can be identified by the XRD peak at about 30.4° [44,45,46]; accordingly, it was found in the three samples in Figure 2b that only the sample annealed at 600 °C (the red XRD curve) had a ferroelectric crystal structure. In contrast, on the yellow curve (annealed at 500 °C), the peak at about 26.4° is believed to be derived from the graphene (002) phase [47]; on the blue curve (annealed at 700 °C), neither the o-(111) phase peak nor the graphene (002) peak can be found. These XRD results indicate that 600 °C is an optimized annealing temperature for fabricating ferroelectric HZO film. Furthermore, we also examined the XRD spectra of HZO films on the bare Si substrate (HZO/Si) at 500 °C, 600 °C, and 700 °C, in which no o-(111) phase could be found on any of them; there were only the monoclinic phases of m-(-111) and m-(111) (provided in Figure S2). By comparing HZO/GO/Si (700 °C) with HZO/Si (700 °C), the absence of monoclinic XRD peaks can be found in the blue curve (Figure 2b). We think this discrepancy might be caused by the limited temperature tolerance of the graphene coating, which is about 600 °C. When the annealing temperature is 700 °C, the GO film will be damaged, so neither the monoclinic phases nor the o-(111) phase can be formed on the HZO film; meanwhile, the graphene (002) cannot be found in the blue curve.

Moreover, these XRD examinations also suggested that GO played an important role in fabricating the total solution-processed ferroelectric HZO film; we suppose there may be some interfacial interactions between the Hf or Zr ions in HZO and the oxygen atoms in GO. The mechanism of this GO-aiding function is illustrated in Figure 2a and was tested by X-ray photoelectron spectroscopy (XPS), which was conducted on the HZO/GO/Si and GO/Si samples. The core spectra of the main elements (C, O, Hf, and Zr, indicated by the wide spectra in Figure S3) are presented in Figure 2c–f. First, Figure 2c indicates that the main carbon atoms in GO’s honeycomb network structure (i.e., C-C at 284.8 eV) are almost unchanged by the deposited HZO, but the terminal carbon states in GO (i.e., C-O at 286.0 eV and C=O at 288.79 eV) are decreased and disappear after coating HZO. From these changes in carbon chemical states, it is inferred that GO’s aiding function for forming the o-(111) phase may come from the interfacial oxygen-containing groups in GO. Then, further evidence was found in the O 1s core spectra (Figure 2d), which includes the following: (1) the left-shifted contour of HZO/GO/Si (by about 2.1 eV) indicates the appearance of oxygen bonds with Hf or Zr (marked as Hf/Zr-O peak at 530.1 eV in the lower plot of Figure 2d) [48]; (2) the dramatically reduced C=O (at about 532.22–531.83 eV) and C-OH (at about 533.10–533 eV), as well as the disappeared oxygen states in O-C=O (531.2 eV) and C-O-C (533.87 eV), suggest that these vanished oxygen atoms participate in the formation Hf/Zr-O bonds. These observed variations in carbon and oxygen chemical states can also be confirmed by the quantitative analyses in Figure 2c,d (provided in Tables S1 and S2). Third, in Figure 2e, the two characteristic peaks located at 16.9 eV and 18.5 eV are the Hf 4f_7/2_ and Hf 4f_5/2_ orbital energy levels in the Hf-O bond [48], respectively. In Figure 2f, the characteristic peaks located at 182.3 eV and 184.7 eV correspond to the orbital energy levels of Zr 3d_5/2_ and Zr 3d_3/2_ in the Zr-O bond [48], respectively. These results provide proof of the formation of Hf/Zr-O bonds in Figure 2d. The XPS analyses demonstrate that during the proposed processes of total solution-processed HZO film, there is interfacial oxygen competition between Hf or Zr atoms and carbon atoms in GO (illustrated in Figure 2a); the shaping of Hf-O and Zr-O bonds may cause lattice stresses or local distortions in HZO, thereby promoting phase transitions in the crystal structure and the formation of an orthorhombic phase (i.e., o-(111)) [17]. In contrast, for the HZO film on the naked Si substrate (HZO/Si), due to the absence of GO, the Hf-Si and Zr-Si bonds are necessary to firmly coat HZO on Si, and their existence is demonstrated by XPS in Figure S4. We deduce that these Si-mediated interfacial states (without oxygen) may introduce foreign atoms (Si) to HZO and lead to the failure of o-(111) in HZO/Si (proven by XRD in Figure S2). In summary, the aiding function of GO in forming ferroelectric HZO through the total solution process can be clarified experimentally and theoretically. Subsequently, using the same solution process as mentioned above, samples of flexible mica-supported HZO (referred to as HZO/GO/mica) were fabricated and investigated by means of XRD, scanning electron micrograph (SEM), and energy-dispersive X-ray spectroscopy (EDS) mapping. First, the XRD results in Figure 2g indicate there are XRD diffraction peaks of mica substrates and GO for all samples. The o-(111) diffraction peak can only be observed on the red curve, which includes the samples that were spin-coated three, four, and five times. The SEM image of the cross-section of HZO/GO/mica in Figure 2h demonstrates the layered architecture; its uppermost surface region (the red dashed line box marked in Figure 2h) was examined by EDS mapping (shown in Figure 2i), which demonstrated that the main elements are C, O, Hf, and Zr. The spatial distributions of these elements are provided in Figure 2j–m; the uniformly distributed O, Hf, and Zr elements demonstrated that the deposited HZO film is uniform. Meanwhile, the emergence of C elements caused by the superficial EDS mapping also indicates the deposited HZO film is ultra-thin. We tried to measure this thickness but failed. We estimated the thickness of the HZO film to be less than 10 μm based on Figure 2h.

### 3.2. Basic Electric Features and Simulation of Neuro-Responses 

**Figure 3:**
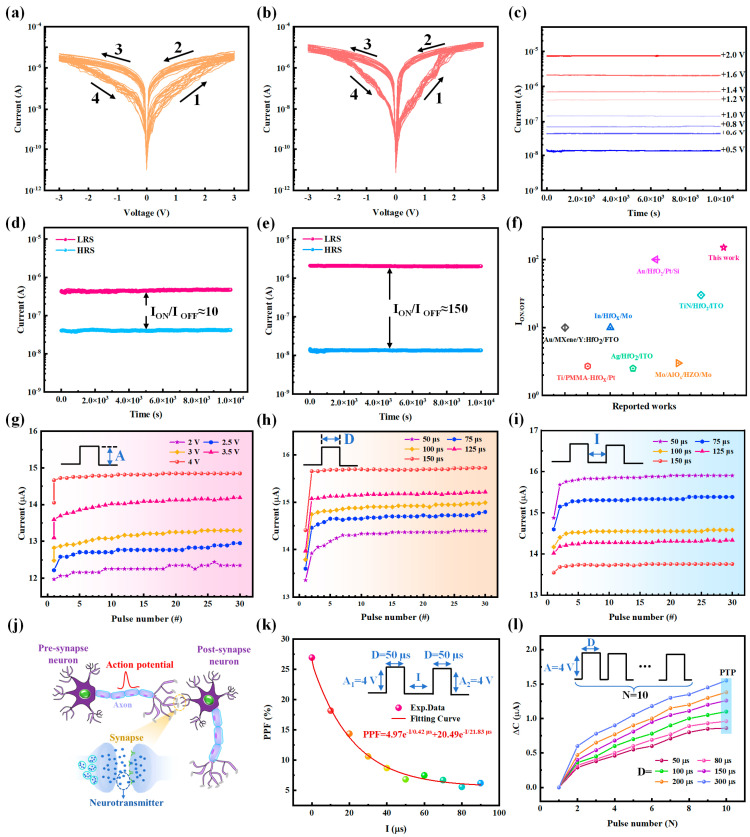
The basic electronic performance of the as-prepared Pt/HZO/GO/Mica memristors. (**a**,**b**) *I-V* curves of 20 cyclic voltametric measurements of devices with HZO films created with 3- and 4-time spin-coating, respectively. (**c**) Multi-state retention properties of a device with 4-time spin-coated HZO films. The bias voltage is controlled from 0.5 V to 2 V. (**d**,**e**) The ON/OFF-state currents of devices with 3- and 4-time spin-coating, respectively. Read voltage is 0.5 V. (**f**) Ratios of ON/OFF-state currents in comparison with previously reported works of hafnium-based memories prepared by solution-processed methods. (**g**–**i**) Pulse responses for different amplitudes (A = 2, 2.5, 3, 3.5, 4 V), durations (D = 50, 75, 100, 125, 150 μs), and intervals (I = 50, 75, 100, 125, 150 μs). (**j**) Schematic diagram of the neural synapse. (**k**) PPF simulation of the as-prepared device. The A of the applied pairs of voltage pulses is 4 V; D is 50 μs; and I = 0, 10, 20, 30, 40, 50, 60, 70, 80, 90 s. PPF (%) = (C_2_ − C_1_)/C_1_ × 100%. (**l**) PTP simulation of the as-prepared device. The high-frequency stimuli pulses (N = 1–10, A = 4 V) have different D values (D = 50, 80, 100, 150, 200, and 300 μs). ∆C = C_N_ − C_1_.

HZO-based flexible memristors fabricated by the total solution processes described in Figure 1b were tested to evaluate their electrical performance as ferroelectric memristors, including the typical *I-V* characteristics, multi-state retention properties, resistance switchable characteristics, and pulse responses. The electric features were measured by using the testing mode depicted in the bottom left of Figure 1b; i.e., the bias voltage is applied on the top electrode (Pt), and the bottom electrode (GO) is grounded. Figure 3a,b show 20 cyclic voltametric *I-V* curves of devices with three- and four-time spin-coating HZO films, respectively. The device with the five-time spin-coating HZO film was also measured, but its I-V feature was abnormal. The resistance switchable characteristic could be easily identified by the varied current with cyclic scanning voltage; the scanning cycle includes four parts, which are labeled as 1 to 4 in Figure 3a,b. In parts 1 and 3, when the absolute bias voltages were scanned from 0 to 3 V, the currents were increased from the lowest points (about 10^−12^ A) to relatively high values (5 × 10^−6^ A and 1 × 10^−5^ A in Figure 3a,b, respectively); conversely, in parts 2 and 4, the currents decreased. These current changes indicate that the resistances of the as-prepared solution-processed HZO memristors are switched between a high resistance state (HRS) and a low resistance state (LRS), and the currents in HRS and LRS are referred to as I_OFF_ and I_ON_, respectively. Meanwhile, the multilevel current states of the device with the four-time spin-coating HZO films were also measured when bias voltages were controlled from 0.5 V to 2 V; the transient currents are plotted in Figure 3c. It can be seen that the currents at each of the levels have a retention time of up to 1 × 10^4^ s and almost no fluctuation. The above experimental phenomenon is derived from the theory of polarization reversal process, which is as follows: the resistance change of the ferroelectric HZO memristor is determined by the ferroelectric domain dynamics, and the different resistance states depend on the degree of ferroelectric domain reversal [4,49]. For the as-prepared HZO-based memristor, the initial polarization directions of the ferroelectric domains are random; there is a barrier effect between the adjacent domains that hinders the carriers’ transportation through the HZO film. So, for the lower-bias voltage, the applied electric field is not strong enough to overcome the internal ferroelectric polarity, and the current is at a low level (i.e., I_OFF_). When the bias voltages are increased over the threshold points, they tend to be consistent with the externally applied electric field; thus, the barrier effect of the domain walls on the carriers’ transportation along the applied electric field is weakened, and the current flow through the HZO-based memristor is gradually increased to a high level (i.e., I_ON_).

The I_ON_/I_OFF_ ratio is an important indicator for evaluating the memory ability of memristors; according to the I_ON_ and I_OFF_ currents of the tested devices presented in Figure 3d,e, the I_ON_/I_OFF_ ratios could be calculated as shown in the insets of these figures. The I_ON_/I_OFF_ of the device with a four-time spin-coated HZO (150) is significantly higher than that of the device with a three-time spin-coated HZO (10). Therefore, the device with a four-time spin-coated HZO film is used in the following experiments. Moreover, the good memory features of the proposed HZO flexible memristor can be testified by comparing the I_ON_/I_OFF_ with the reported hafnium-based memristors that were fabricated using the solution-processed methods [38,50,51,52,53,54,55]. The data plotted in Figure 3f indicate that the I_ON/OFF_ of this work is located at the upper-right corner. Along with the long-term stable multilevel currents in Figure 3c, the as-prepared memristor has the ability of multi-state storage [56]; subsequently, we examined the proposed memristor’s synaptic plasticity and emulated the learning and memory processes by using it [57].

Synaptic plasticity in biology states that the strength of synaptic connections between neurons can be dynamically changed. Figure 3j depicts an example of a neuro-connection between pre- and post-synapse neurons; the intensity of neuro-information transmitted between them can be mediated by a neurotransmitter. Herein, we simulate this synaptic plasticity by using the as-prepared Pt/HZO/GO/mica memristor, in which the top electrode serves as the pre-synapse neuron, and the bottom electrode acts as the post-synapse neuron. The applied signals were applied to the electrodes to simulate their biological action potential, and the current through the memristor was altered; this stimulus-evoked current is called an excitatory postsynaptic current (EPSC). Here, the peak values of the EPSC in responses to 30 continuously varied pulse voltage signals are measured and plotted in Figure 3g–i; the only differences in these pulse-responding tests were the varied pulse amplitude (A), duration (D), and interval (I). First, it can be seen in Figure 3g that the intensities of the EPSCs were enhanced by increasing A from 2 to 4 V (D and I are 100 μs), and for the weak pulses (like A = 2, 2.5 V), more pulses were needed to generate stable EPSCs (i.e., the saturation parts of the curves: A = 3.5 and 4 V). These results indicate that synaptic plasticity-to-action potentials with different intensities can be simulated by the as-prepared device. Next, the action potential duration’s impacts on synaptic plasticity were mimicked by varying D from 50 to 150 μs (A = 4 V and I = 50 μs). The up-shifted EPSC curves in Figure 3h indicate that the simulation of stronger neural responses evoked by the prolonged action potential is fulfilled, and more pulses are needed to make the EPSC saturated. Third, the interval between two adjacent action potentials is also an influencing factor in biological synaptic plasticity; i.e., the memory for the action potential tends to attenuate after the action is withdrawn. Then, if the next action potential is delayed, the neural response will be decreased. The down-shifted curves in Figure 3i, by increasing I from 50 to 150 μs (A = 4 V and D = 100 μs), show that the as-prepared memristors’ EPSCs can mimic this weakening synaptic plasticity caused by the delayed action potentials. In summary, these pulse-responding results indicate that pulse-controllable EPSCs are beneficial for simulating synaptic plasticity. Thus, the as-prepared devices were used to simulate two neurobiological responses (PPF [39,40] and PTP [39,41,42]). PPF, which refers to the superposition of synaptic weights resulting from two consecutive stimuli, is essential to perception and memory in the nervous system. To validate the as-prepared device’s ability to mimic PPF, the simulation tests were performed by applying pairs of voltage pulses (D = 50 μs and A= 4 V, depicted by the inset in Figure 3k); the devices’ currents were measured, and the current peak values inspired by the first and second pulses are referred to as C_1_ and C_2_, respectively. Their change ratios ((C_2_ − C_1_)/C_1_ × 100%) with the prolonged I were calculated and denoted as PPF; the experimental data points (PPF, I) are plotted in Figure 3k. Additionally, they were well fitted in a double exponential decay function [40], which is mathematically expressed by the red formula in Figure 3k. The fitting results indicate that the responses of the as-prepared device to two consecutive pulses can be deduced with the increased I and exhibit fast (0.42 μs) and slow (21.83 μs) exponential decay trends; these simulated results are in agreement with the description in reference [54]. The other factor, PTP, is a phenomenon of high-frequency stimulus-induced synaptic weight enhancement [41]; to simulate it using the as-prepared devices, the currents after exposure to high-frequency stimuli (the pulses depicted in the inset of Figure 3l) were measured, where the amplitude of pulse A was controlled at 4 V, and the number (N) of pulses in a controlled time varied from 1 to 10. The current peaks after experiencing the first and Nth pulses are denoted as C_1_ and C_N_, and ∆C is C_N_ minus C_1_; the experimental data for the varied N and the different pulse width, D, are plotted in Figure 3l. It can be seen that for each of the constant D values, the ∆C data increase with increasing N, which means that the memristor successfully simulated high-frequency stimulus-induced synaptic weight enhancement in biological synapses [42]. Furthermore, with an increasing D (50–300 μs), the data curves in Figure 3l are shifted upward, which means that the artificial synaptic currents were enhanced by extending the pulse duration (D); this is also in line with biological synaptic behavior [39].

### 3.3. Mechanical and Thermal Influences

**Figure 4:**
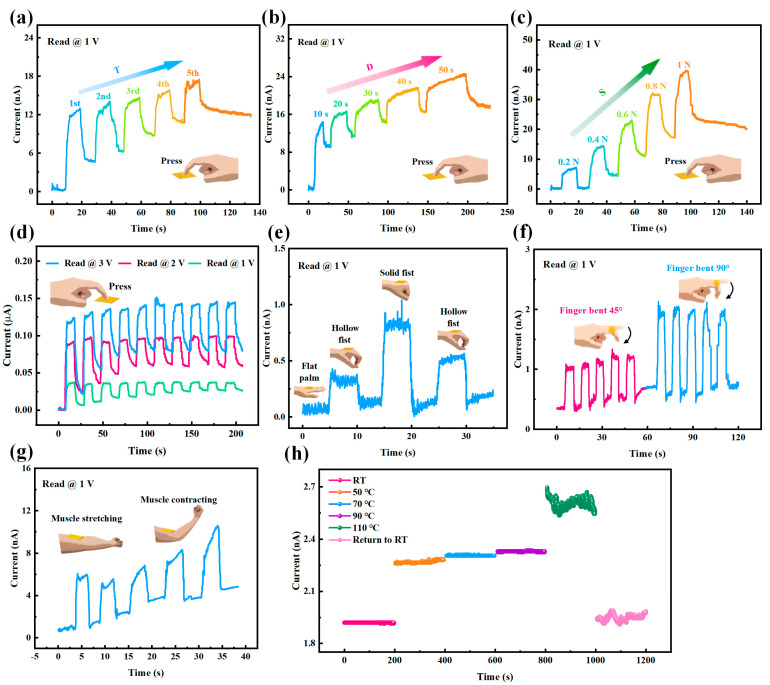
Mechanical and thermal influences of as-prepared memristors. (**a**) The transient current of the as-prepared device when consecutively pressed by a finger five times (T = 1–5) with the same press duration (D = 10 s) and press strength (S = 0.3 N). Read @ 1 V. (**b**) The transient current of the as-prepared device when consecutively pressed by a finger five times with different press durations (D = 10–50 s) and same press strength (S = 0.3 N). Read @ 1 V. (**c**) The transient current of the as-prepared device when consecutively pressed by a finger five times with different press strengths (S = 0.2–1 N) and same press duration (D = 10 s). Read @ 1 V. (**d**) The transient currents of the as-prepared device were measured at different read voltages (Read @ 1–3 V) with the same press strength (S = 1 N), press duration (D = 10 s), and press times (T = 10). (**e**) The transient current of the device varies with hand position (flat palm, hollow fist, and solid fist) when the device is placed on the back of the palm. Read @ 1 V. (**f**) The transient current of the device varies with finger bending (finger bend at 45° and 90°). Read @ 1 V. (**g**) The transient current of the device varies with brachialis muscle stretching and contracting. Read @ 1 V. (**h**) The measurement of the temperature-sensitive characteristics of the as-prepared device. The device was placed on a heated platform and tested at room temperature (RT), 50 °C, 70 °C, 90 °C, and 110 °C and returned to RT. Read @ 2 V.

The above-mentioned electrical features demonstrate that the as-prepared Pt/HZO/GO/Mica memristors possess excellent multi-state resistance switching abilities and synaptic plasticity, which may provide powerful support for constructing multistate memory and artificial neural networks. However, for a flexible device, we hesitate about whether it can suffer from environmental variations, such as deformation and temperature changes. Accordingly, we conducted mechanical and thermal influence tests. First, the as-prepared flexible HZO memristor was pressed by a finger, as depicted by the inset in the bottom right of Figure 4a; the force of each pressing was about 0.3 N, and the duration of each press was 10 s. The transient current during five consecutive presses was measured at 1 V bias voltage and the room temperature (about 27 °C); the data are plotted in Figure 4a. It was found that the current increased and fell in agreement with each time (T) of the presses and increased to a high level after five presses, i.e., from about 0.14 nA to 12.1 nA. We deduce that this pressure-induced current variation is due to the piezoelectric effect of the HZO film, which is the movement of oxygen ions within the lattice in the HZO film caused by the external pressure, resulting in increased electric polarization and thereby generating an enhanced current [58].

Second, it was also found that the press-elevated current depends on the duration (D) of each pressing; when D increased from 10 s to 50 s, the transient current was measured under the same working conditions mentioned above (as plotted in Figure 4b). It had a similar contour profile as in Figure 4a, but the amplitudes and widths of each pulse were different: (1) The current grew close to 14.4 nA for the first pressing (D = 10 s), and after five presses, it stayed at about 17.9 nA; that is to say, the overall current level in Figure 4b is higher than in Figure 4a. (2) The extended widths of the current pulses can be identified by comparing Figure 4a,b, and they were also enlarged in agreement with the prolonged D from 10 s to 50 s. Third, the dependence of current on the strength (S) of the press was examined under the same working conditions as in Figure 4a and with D = 10 s; the plotted transient current in Figure 4c demonstrated that the current of the as-prepared HZO-based memristor could respond to the intensified tapping forces, as shown by the gradually raised current pulses from S = 0.2 N to S = 1 N (in Figure 4c). We think that the increased D and S caused current increases and current pulse widening, which are consistent with the pressure-induced ferroelectric polarization enhancement [58,59]. Additionally, the stability of the press-induced current change was also demonstrated by the repetitively measured transient currents provided in Figure S5.

Furthermore, we also found that the amplitudes of current pulses induced by similar finger pressing (S = 1 N and D = 10 s) were amplified when the bias voltages were increased from 1V to 3 V, as evidenced by the transient currents in Figure 4d. It can be seen that for the same pressing, the amplitudes of the pressing-caused current pulses were amplified with the bias voltages increasing from 1 V to 3 V; this is in accordance with the basic I-V features provided in Figure 3a. We think the reason for the increased current responses may be explained from two points: (1) under higher-bias voltage, ferroelectric polarization is strengthened, and then, the carrier’s transportation through HZO film may be facilitated, and the current can be elevated; (2) at the same time, the electric field across the HZO film may also be increased by higher-bias voltage, which also helps to increase current. Furthermore, for the higher-bias voltage, since the ferroelectric polarities in the HZO film have been excited, the residual unpolarized parts are smaller; thus, the pressure-induced influence on ferroelectric polarization, which is mentioned above in the explanation of Figure 4a, will be smaller, and the pressure-caused current changes will be easier to saturate. This deduction is in accordance with the measured transient currents in Figure 4d; the current pulses of higher-bias voltages (2 V and 3 V) tend to saturate after three presses, while for the lower bias voltage (1 V), the current pulses are gradually saturated after seven presses. Furthermore, it can also be seen that the pressure-induced currents were all elevated to higher levels after the pressure was withdrawn, which indicates that the as-prepared HZO-based memristor can not only respond to the pressure but also remember it.

Other kinds of mechanical tests were also conducted by measuring the transient current changes caused by deformations that were generated by the motions of the hand, finger, and brachialis muscle, as depicted in the insets of Figure 4e–g, in which the as-prepared HZO-based memristor was worn on the palm back, the finger joint, and the upper arm. The transient current was measured under a bias voltage of 1 V and plotted. In Figure 4e, it can be seen that the current pulses were generated by the palm opening and closing, which is shown by the insets depicting a flat palm, a hollow fist, a flat palm, and a solid fist. Meanwhile, an entire test video is provided in Supplementary Material Video S1. The transient current in Figure 4f indicates that the current pulses were generated by the finger bending, and the amplitudes of the pulses were enhanced with bending angles of 45° to 90°. Moreover, it was found that the transient current was also affected by the brachialis muscle stretching and contracting, as shown in Figure 4g. All these mechanical test results clearly demonstrate that the as-prepared flexible HZO-based memristor is sensitive to mechanical disturbances, like pressure (Figure 4a–d), deformation (Figure 4e–g), and airflow from an ear wash ball (Figure S6). In particular, it can have memories for these disturbances, which makes it a good candidate as an artificial tactile synapse.

Furthermore, we examined the influence of temperature on the as-prepared HZO-based memristor. It was found that when the temperature was gradually increased from room temperature (RT, about 27 °C) to 110 °C, the current was elevated, but for the temperature of 110 °C, the current became unstable; meanwhile, it was also found that the device returned to the initial current level, but still with noise, when it was cooled to RT again, as shown in Figure 4h. We deduce that this temperature-induced current change may be attributed to the GO’s conductivity and the unstable interface states (Hf-O and Zr-O) between HZO and GO, which were demonstrated by XPS in Figure 2e,f. That is to say, when temperature is increased in the lower range, more carriers can be excited in the GO layer, thereby enhancing the current (the pink, orange, blue, and purple parts in Figure 4h); for 110 °C (green part in Figure 4h), the interfacial bonding states (Hf-O and Zr-O in Figure 2e,f) may be weakened due to the high temperature, leading to current instability, and this effect was still present when the temperature gradually cooled to RT (the light pink part in Figure 4h). This deduction may be experimentally supported by the unchanged current of the similar device (without the GO layer) in Figure S7.

The above-mentioned mechanical and thermal influence tests of our flexible Pt/HZO/GO/mica memristor further explore its performance under environmental changes. Mechanically, the memristor’s transient current varied with pressing time, duration, strength, and bias voltage. Deformations from hand, finger, and muscle movements also induced current changes. These mechanically related current variations stemmed from the piezoelectric and ferroelectric polarization effects of the HZO film. Thermally, as the temperature increased from 27 °C to 110 °C, the current first rose and then became unstable due to GO’s conductivity and the HZO-GO interface bonding state changes. Overall, environmental changes significantly impact the memristor’s performance, which is critical for its application in advanced wearable electronics.

### 3.4. Application for Morse Code Recognition

**Figure 5:**
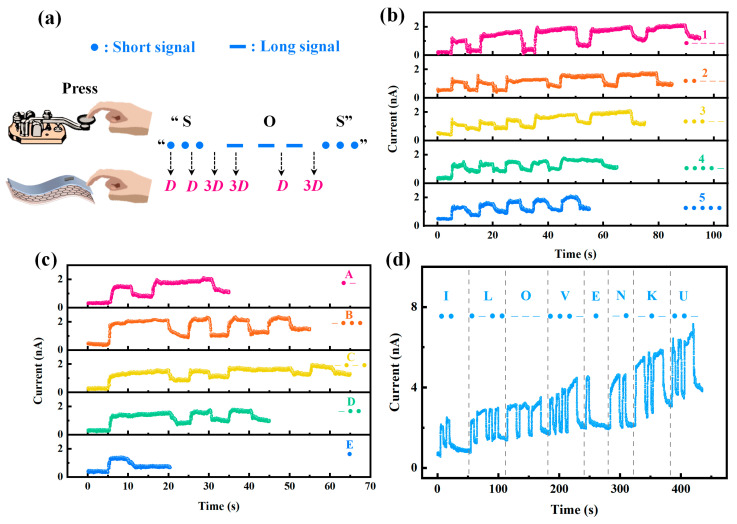
Application for Morse code recognition. (**a**) Schematic diagram of a finger pressing down on the prepared device in analogy to a telegraph sending out Morse code. The dots represent short signals, and the dashes represent long signals. The black arrows point to the duration, *D*, of the press and pause for the example “SOS” code. We were able to recognize the Morse code values of the (**b**) numbers “1, 2, 3, 4, 5”; (**c**) letters “A, B, C, D, E”; and (**d**) phrase “I LOVE NKU”. The pressure of the finger press is 0.1 N. Read @ 1 V.

Building on the results from the mechanical influence tests, our flexible HZO-based memristor revealed remarkable adaptability to environmental changes, prompting us to explore its potential applications. The device’s excellent pressure properties, a key finding from these tests, opened up an avenue for exploring Morse code recognition. Morse code uses specific sequences of short signals (dots) and long signals (dashes) to represent letters, numbers, and punctuation marks. Although traditional Morse code operations are also pressure-based manual manipulations, the associated devices are typically rigid. In contrast, our flexible HZO-based memristor can be easily integrated into a wide variety of environments. This flexibility allows for more versatile usage scenarios, such as being incorporated into wearable devices or conforming to irregular surfaces. As depicted in Figure 5a, we applied 0.1 N of pressure with finger presses and set specific rules for the pressing durations and pauses. For instance, we defined the duration, *D*, of dots as 5 s, the duration of dashes as 3*D*, the pause between dots and dashes within a single character as *D*, the pause between characters as 3*D*, and the pause between words as 7*D*. By carefully adjusting the pressing durations in accordance with these rules, we managed to recognize various Morse code elements. As shown in Figure 5b–d, we successfully recognized the numbers “1, 2, 3, 4, 5”; the letters “A, B, C, D, E”; and the phrase “I LOVE NKU”. The device precisely detected the pressure-based signals and translated them into their respective Morse code representations, and could even memorize this information. Combined with its storage capacity, this device unlocks new applications in tactile sensing, silent communication, and real-time Morse code data processing.

## 4. Conclusions

In this work, we developed a flexible Pt/HZO/GO/mica memristor via the total solution-processed method, which has effectively addressed several key challenges in the field of ferroelectric memristors. The formation of the HZO ferroelectric phase is critically dependent on the interfacial oxygen competition mechanism between the HZO layer and the GO bottom electrode. The resulting device demonstrates remarkable electrical properties, with a switching ratio of around 150 and the ability to stably maintain eight distinct resistance levels, thereby fulfilling the requirements of multi-state storage. It can also effectively simulate neural responses such as PPF and PTP, indicating its potential in neuromorphic computing. By combining the ferroelectric polarization principle and the piezoelectric effect of HZO and the influence of GO, the device under mechanical (e.g., deformations caused by finger presses, joint movements, and muscle contractions) and thermal (27 °C to 110 °C) influences has exhibited remarkable performance. Notably, Morse code was successfully recognized using the pressure properties of the device. The ability of the device to accurately convert and store information opens up new possibilities for non-volatile memory applications in silent communication and provides a strong impetus for the development of wearable electronics. Future efforts can focus on optimizing device performance and exploring more functions based on this design.

## Data Availability

Further inquiries can be directed to the corresponding author.

## Supplementary Materials

The following supporting information can be downloaded at https://www.mdpi.com/article/10.3390/s25206429/s1: Figure S1: The XPS core spectra of (a) C 1s and (b) O 1s for graphene-conductive coatings not treated with 280 °C high temperature; Figure S2: XRD results of HZO/Si at different annealing temperatures (500 °C, 600 °C, and 700 °C); Figure S3: XPS wide spectra of (a) GO/Si and (b) HZO/GO/Si structures; Figure S4: XPS core spectra analysis of Zr and Hf element in HZO thin films on bare silicon substrates; Figure S5: Transient current of memristors under force application; Figure S6: Transient current in memristors under blowing conditions; Figure S7: The thermal tests of the as-prepared HZO-based memristors; Table S1: XPS analysis of the C 1s spectra of the GO/Si and HZO/GO/Si samples; Table S2: XPS analysis of the O 1s spectra of the GO/Si and HZO/GO/Si samples; Video S1: Current changes in hand movements.
