# Supplementary material for: Total Solution-Processed Zr: HfO2 Flexible Memristor with Tactile Sensitivity: From Material Synthesis to Application in Wearable Electronics
Source: Sensors (Basel). 2025 Oct 17;25(20):6429. doi: 10.3390/s25206429 (PMC12567743; doi:10.3390/s25206429)
Supplement: Supplementary file 1 [file sensors-25-06429-s001.zip › sensors-3908134-supplementary_author-proof-done/Sensors_Supplementary information.pdf]

Supplementary information

Total Solution-processed Zr: HfO_2 Flexible Memristor with Tactile Sensitivity: From Material Synthesis to Application in Wearable Electronics

Luqi Yao, and Yunfang Jia *

College of Electronic Information and Optical Engineering, Nankai University, Tianjin 300071, China

*Corresponding author. E-mail: jiayf@nankai.edu.cn (Y. Jia).

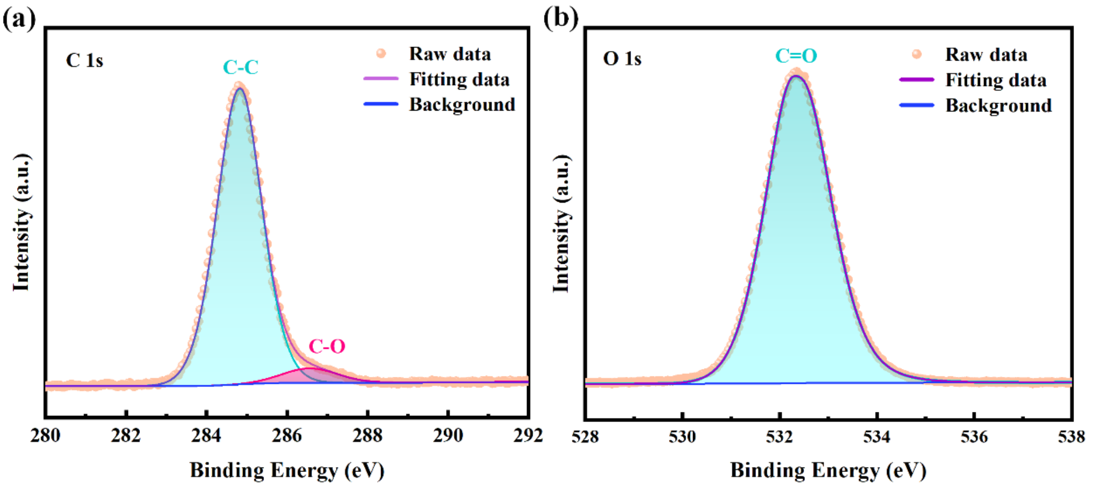

Figure S1. The XPS core spectra of (**a**) C 1s and (**b**) O 1s for graphene-conductive coatings not treated with 280 °C high temperature. Compared to graphene oxide (GO) cured at 280 °C, the untreated graphene has fewer oxygen-containing groups, so the graphene conductive coatings form GO under the 280 °C high-temperature treatment.

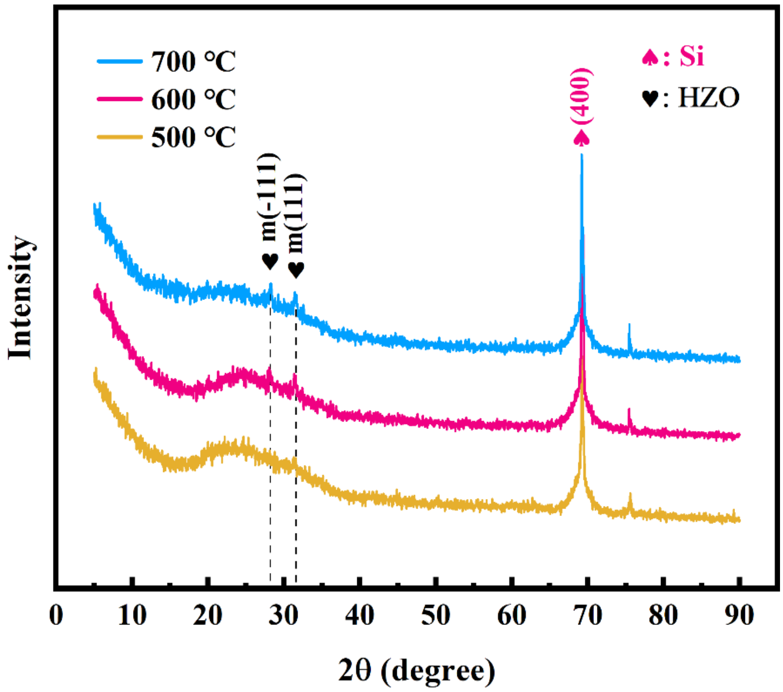

Figure S2. XRD results of HZO/Si at different annealing temperatures (500 °C, 600 °C, and 700 °C). In the XRD curves for these three annealing temperatures, only the monoclinic phase at 28.2° (m (-111)) and 31.5° (m (111)) is observed.

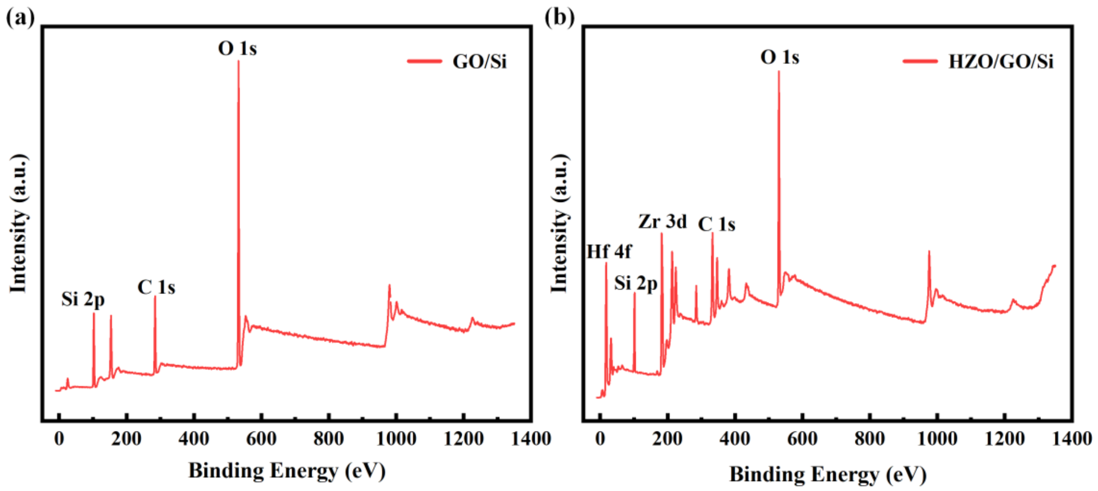

Figure S3. XPS wide spectra of (**a**) GO/Si and (**b**) HZO/GO/Si structures.

The XPS results of the GO/Si and HZO/GO/Si samples were analyzed by the *Avantage* software using a *Gaussian–Lorentzian* mixing fitting method, and the results are shown in Tables S1 and S2. Among them, peak area (P) denotes the un-normalized spectral peak area, and peak area (N) denotes the normalized spectral peak area. The relationship between them can be derived from the following equation:

$$\text{Peak Area (N)} = \text{Peak Area (P)} / (\text{SF} \times \text{TXFN} \times \text{ECF}) \quad (1)$$

where TXFN is transfer function, SF is sensitivity factor, and ECF is energy compensation factor.

By comparing the peak area (N) and the atomic concentration ratios of each peak for the two samples, it can be observed that the oxygen-containing groups of both the C 1s and O 1s spectra are decreasing.

Table S1. XPS analysis of the C 1s spectra of the GO/Si and HZO/GO/Si samples.

Samples	Peaks	Binding Energy (eV)	Peak Area (P) CPS. eV	Peak Area (N) TPP-2M	Atomic (%)	Groups
**GO/Si**	C 1s	284.8	18003.88	0.41	80.58	C-C
		286.05	3251.92	0.07	14.56	C-O
		288.79	1084	0.02	4.86	C=O
**HZO/GO/Si**	C 1s	284.8	2823.97	0.65	90.34	C-C
		286	3070.73	0.07	9.66	C-O

Table S2. XPS analysis of the O 1s spectra of the GO/Si and HZO/GO/Si samples.

Samples	Peaks	Binding Energy (eV)	Peak Area (P) CPS.eV	Peak Area (N) TPP-2M	Atomic (%)	Groups
**GO/Si**	O 1s	531.20	9359.97	0.08	7.08	O-C=O
		532.22	97881.02	0.83	74.05	C=O
		533.10	21426.71	0.18	16.22	C-OH
		533.87	3505.09	0.03	2.65	C-O-C
**HZO/GO/Si**	O 1s	530.10	139394.13	1.18	81.16	Hf/Zr-O
		531.83	27801.80	0.23	16.20	C=O
		533	4537.66	0.04	2.64	C-OH

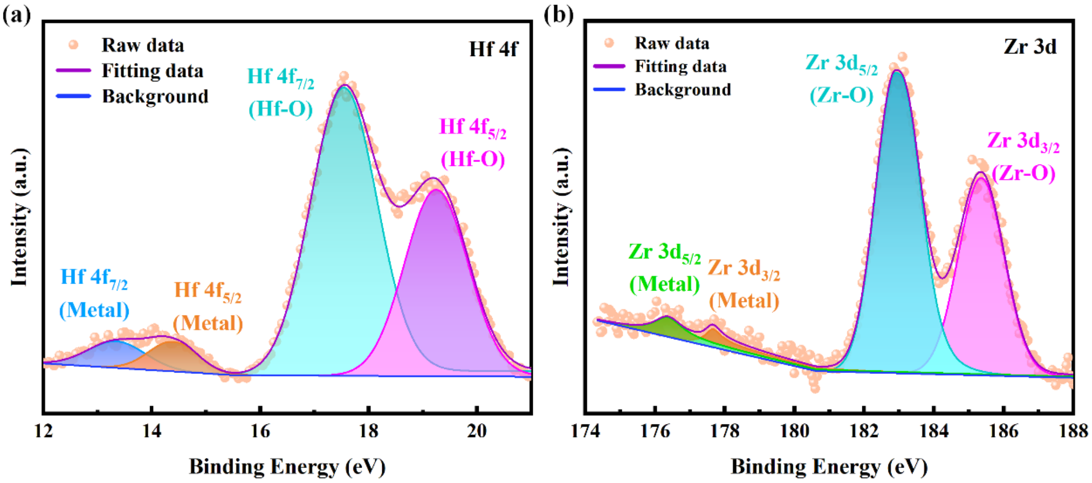

Figure S4. XPS core spectra analysis of Zr and Hf element in HZO thin films on bare silicon substrates. (**a**) The XPS core spectra analysis of the Hf element of HZO film deposited directly on bare silicon substrate shows that the characteristic peaks located at 17.54 eV and 19.24 eV correspond to the Hf $4f_{7/2}$ and Hf $4f_{5/2}$ orbital energy levels of the Hf-O bond, respectively, and the characteristic peaks at 13.35 eV and 14.38 eV correspond to the Hf $4f_{7/2}$ and Hf $4f_{5/2}$ orbital energy levels of the Hf-Si bond, respectively. (**b**) The XPS core spectra analysis of the Zr element of HZO film deposited directly on bare silicon substrate shows that the characteristic peaks located at 182.91 eV and 185.36 eV correspond to the Zr $3d_{5/2}$ and Zr $3d_{3/2}$ orbital energy levels of the Zr-O bond, respectively, and the characteristic peaks at 176.38 eV and 177.67 eV correspond to the Zr $3d_{5/2}$ and Zr $3d_{3/2}$ orbital energy levels of the Zr-Si bond, respectively.

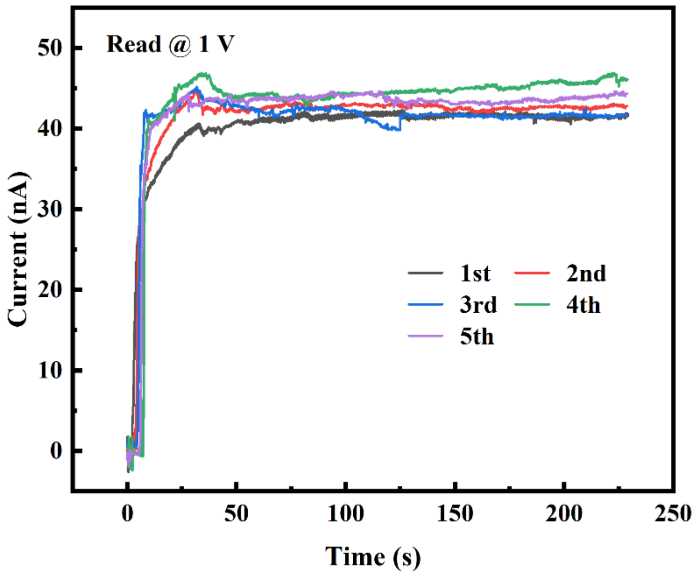

Figure S5. Transient current of memristors under force application. The repetitively measured transient currents when the 1 N pressure was exerted on the as-prepared HZO-based memristor. The bias voltage was controlled at 1 V; the same measurement was repeated five times; and the current curves are plotted in the colors of black, red, blue, green, and purple. It can be seen that the five curves are almost overlapping, which means the influence of pressure on the proposed device is stable if the other working conditions, like bias voltage and environmental temperature, are constant.

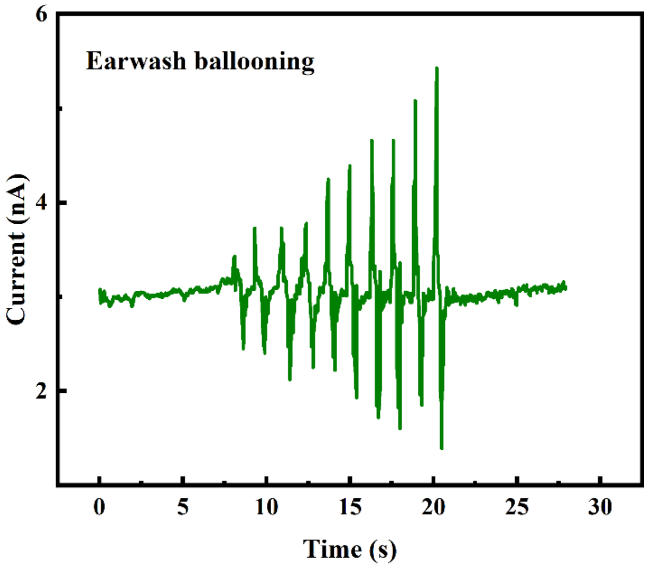

Figure S6. Transient current in memristors under blowing conditions. The transient currents were measured when the as-prepared device was fixed on a horizontal tabletop and blown vertically 10 times with an ear wash ball. The test conditions were at 1 V bias voltage and room temperature.

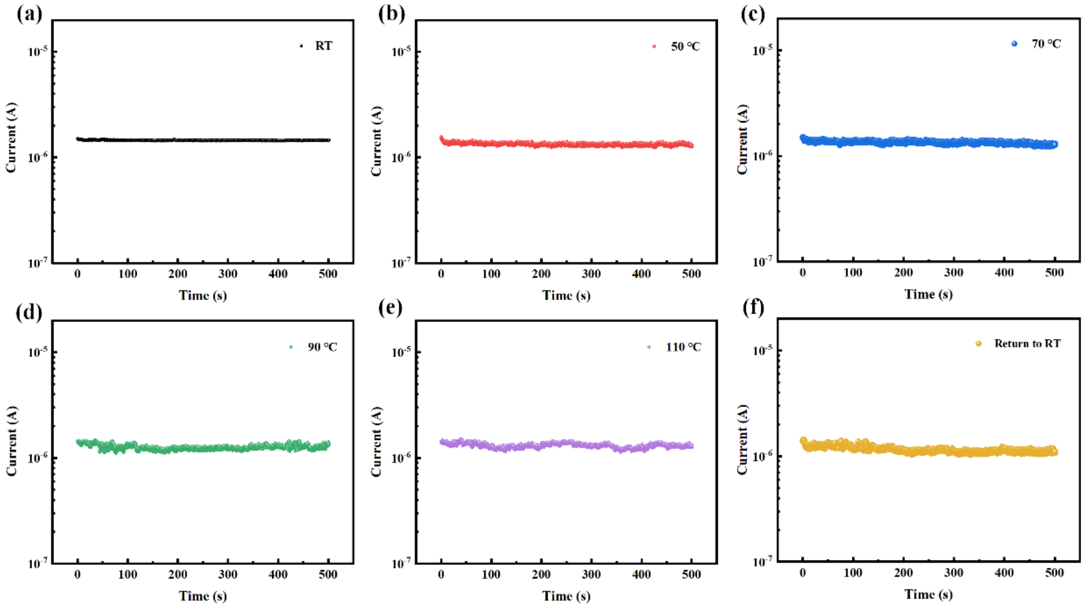

Figure S7. The thermal tests of the as-prepared HZO-based memristors. The device was placed on a heated platform to control its environmental temperature. Its transient currents were measured at a constant bias voltage (1 V) and at different temperatures from low to high, i.e., room temperature (RT, in (**a**)), 50°C (**b**), 70°C (**c**), 90°C (**d**), and 110°C (**e**) and then remeasured when it was gradually cooled to return to RT (**f**).
